# Age-Related Quality of Life and Psychosocial Impact of Chin Asymmetry in Adolescents and Young Adults Undergoing Orthodontic and Orthognathic Correction

**DOI:** 10.3390/healthcare11131855

**Published:** 2023-06-26

**Authors:** Serban Talpos, Marius Pricop, Camelia Szuhanek, Robert Avramut, Nicoleta Nikolajevic-Stoican, Raluca Maracineanu, Roxana Talpos, Tareq Hajaj, Malina Popa

**Affiliations:** 1 Discipline of Oral and Maxillo-Facial Surgery, Faculty of Dental Medicine, “Victor Babes” University of Medicine and Pharmacy Timisoara, Revolutiei Boulevard 9, 300041 Timisoara, Romania; talpos.serban@umft.ro (S.T.); pricop.marius@umft.ro (M.P.); 2 Discipline of Orthodontics, Faculty of Dental Medicine, “Victor Babes” University of Medicine and Pharmacy Timisoara, Revolutiei Boulevard 9, 300041 Timisoara, Romania; cameliaszuhanek@umft.ro; 3 Doctoral School, “Victor Babes” University of Medicine and Pharmacy, Eftimie Murgu Square 2, 300041 Timisoara, Romania; nicoleta_stoican@yahoo.com (N.N.-S.); ralucazibileanu@yahoo.com (R.M.); 4 Discipline of Odontotherapy-Endodontics, Faculty of Dental Medicine, “Victor Babes” University of Medicine and Pharmacy, Eftimie Murgu Square 2, 300041 Timisoara, Romania; roxanaclinci@yahoo.com; 5 Discipline of Prostheses Technology and Dental Materials, Faculty of Dental Medicine, “Victor Babes” University of Medicine and Pharmacy, Eftimie Murgu Square 2, 300041 Timisoara, Romania; tareq.hajaj@umft.ro; 6 Department of Pediatric Dentistry, Faculty of Dental Medicine, “Victor Babes” University of Medicine and Pharmacy, Eftimie Murgu Square 2, 300041 Timisoara, Romania; popa.malina@umft.ro

**Keywords:** quality of life, health-related quality of life, facial asymmetry, psychological stress

## Abstract

Craniofacial asymmetry can have significant psychosocial implications, affecting the quality of life in adolescents and young adults. This study aimed to assess the impact of age and complexity of craniofacial asymmetry on quality of life and psychosocial outcomes in patients undergoing orthodontic and orthognathic correction. A cross-sectional study was conducted on 149 patients aged 13–26 years with moderate or severe craniofacial asymmetry that had a gnathion deviation higher than 2 mm, according to the American Association of Orthodontists. Participants were divided into three groups: teenagers (*n* = 53), adults (*n* = 46), and a control group (*n* = 50) with relative craniofacial asymmetry. Quality of life and psychosocial impacts were evaluated using validated questionnaires that measure health-related quality of life (SF-36), body image satisfaction and self-acceptance (BIQLI), anxiety and depression levels (HADS), and perceived stress (PSS-10). These tools provided an encompassing appraisal of the psychological and social implications associated with craniofacial asymmetry before and six months after orthodontic and orthognathic correction. Before the intervention, adults had higher mental health scores compared to teenagers (*p* = 0.037). At the 6-month follow-up, no significant differences in mental health scores were observed between the two groups (*p* = 0.121). BIQLI results showed significant differences in satisfaction with appearance and self-acceptance between teenagers and adults, both before intervention (*p* = 0.045 and *p* = 0.051, respectively) and at six months (*p* = 0.062 and *p* = 0.031, respectively). HADS results showed significant differences in anxiety levels before intervention (*p* = 0.039) but not at six months (*p* = 0.133). PSS-10 results showed no significant differences in perceived stress between the groups. In conclusion, this study demonstrates that craniofacial asymmetry significantly impacts the quality of life and psychosocial well-being of adolescents and young adults. Specifically, teenagers, as compared to young adults, reported lower satisfaction with their appearance and lower self-acceptance before intervention, underscoring the profound psychosocial challenges that adolescents with craniofacial asymmetry may experience. These age-related differences underscore the importance of tailored interventions to address unique psychosocial needs at different developmental stages.

## 1. Introduction

Craniofacial asymmetry refers to the presence of structural dissimilarities between the two halves of the face and skull, arising from a range of factors, including genetic predispositions, developmental anomalies, and external influences [1,2]. While a certain degree of asymmetry is considered normal and can be observed in the general population, significant asymmetries may pose functional or aesthetic concerns, necessitating medical intervention to improve appearance and functionality [3]. The prevalence of craniofacial asymmetry varies depending on the type and severity of the asymmetry in question. Mild asymmetries are common, with some studies estimating that up to 95% of individuals exhibit some degree of subclinical facial asymmetry [4]. However, more severe cases are less common, affecting approximately 1 in 2000 to 1 in 4000 live births [5].

Craniofacial asymmetry in adolescents and young adults can have a profound impact on quality of life, encompassing both physical and psychosocial aspects [6]. The presence of significant facial asymmetry may lead to functional challenges, such as masticatory difficulties, speech impediments, or temporomandibular joint disorders [7]. Moreover, during this critical developmental stage, self-image and social acceptance are heavily influenced by appearance [8]. Consequently, individuals with craniofacial asymmetry may experience feelings of self-consciousness, social isolation, or stigmatization, which can contribute to increased levels of anxiety, depression, and reduced self-esteem [9]. These psychosocial factors, in conjunction with functional concerns, may motivate patients to seek orthodontic and orthognathic interventions in order to improve their quality of life.

Orthodontic and orthognathic correction can play a significant role in addressing both the functional and aesthetic aspects of craniofacial asymmetry [10]. Treatment may include a combination of orthodontic appliances, such as braces or expanders, as well as orthognathic surgery to realign the jaws and achieve proper occlusion [11]. These interventions improve not only facial symmetry and balance but also facilitate proper function, including chewing, speaking, and breathing [12]. As a result, patients often report significant improvements in their overall quality of life following treatment. However, it is essential to consider the emotional and psychological impact of the treatment process itself, which can be lengthy and challenging for adolescents and young adults. Also, the psychosocial impact of undergoing orthodontic and orthognathic correction should not be underestimated. The treatment process can be demanding and may entail temporary aesthetic and functional compromises, such as the need for orthodontic appliances, temporary restrictions on oral function, and infrequently leaving visible surgical scars. Patients may also experience discomfort or pain during the recovery period [13]. While the treatment process is inherently challenging and may initially exacerbate feelings of self-consciousness due to the temporary aesthetic and functional changes, it can also offer potential psychosocial benefits. The prospect of improved facial symmetry and functionality can foster hope and motivation in patients. Moreover, supportive interaction with healthcare professionals during treatment may serve as an additional source of psychosocial support, helping patients cope with their condition and the demanding treatment process. In addition, treatment may span several years, with multiple appointments and adjustments required to achieve optimal outcomes.

Teenagers and young adults go through a critical developmental period, marked by significant physical, psychological, and social changes. During adolescence and young adulthood, self-image and peer acceptance become increasingly important, heightening the potential psychosocial impact of visible conditions like craniofacial asymmetry [8]. However, a distinction between teenagers and young adults can allow for a nuanced understanding of age-related impacts, as maturity and coping mechanisms may differ between these groups [14]. Furthermore, the literature suggests that teenagers may experience more pronounced psychosocial effects from visible physical conditions due to these developmental factors [15].

The central hypothesis of this research revolves around the understudied relationship between facial asymmetry and psychological disorders, like anxiety and depression. Thus, it is assumed that facial asymmetry has a more substantial negative effect on the quality of life (QoL) in adolescents compared to young adults who share this condition. Furthermore, it is conjectured that teenagers with facial asymmetry have a higher incidence of stress-related disorders relative to their young adult counterparts. The primary objective of this study is to scrutinize and contrast the QoL in adolescents and young adult patients burdened by facial asymmetry by utilizing validated questionnaires to explore the prevalence of stress-related disorders, notably anxiety and depression, amongst these patients. A distinctive aspect of this study involves evaluating these disorders, both before and subsequently to orthognathic intervention, offering a comprehensive understanding of the psychosocial implications of craniofacial asymmetry.

## 2. Materials and Methods

### 2.1. Design and Ethics

A case–control study was designed at the Emergency Clinical Municipal Hospital in Timisoara, Romania, during a three-year span (2020–2023), at the Department of Oral and Maxillo-Facial Surgery. The study was conducted according to the guidelines of the Declaration of Helsinki. The researchers involved in the current study gathered background and medical data from the hospital database and the associated patients’ paper records, where all treatments, procedures, and demographics were registered. It was ensured that all patients involved in the study provided their written informed consent, or their legal counterparts’ agreement for study participation, for patients below the age of legal consent.

### 2.2. Patients’ Selection

The inclusion criteria comprised the following particularities: (1) patients required to be between 13 and 26 years old; (2) patients must have a diagnosis of moderate or severe facial asymmetry of the gnathion, proven by 3D-imaging methods; (3) no previous maxillo-facial interventions; (4) a diagnosis of relative craniofacial asymmetry for the control group. Patients were excluded for the following reasons: (1) no commitment to follow-up at six months after the initial intervention; (2) incomplete medical records; (3) lack of consent; and (4) being younger than 14 or older than 25 years. Although gnathion deviation was considered as the main inclusion criterion and method of patient classification, we did not exclude patients with different asymmetry of the face, unless the severity made it impossible to compare with the other included patients.

The study’s sample size was determined through a method of convenience sampling, which culminated in the identification of 192 ideal cases. This number was calculated taking into account an estimated 10% prevalence rate of craniofacial asymmetry in the general population [16], factoring in a 5% margin of error and maintaining a confidence level of 95%. The set statistical significance threshold was 0.05, and the calculation of statistical power (1-β) was determined to be 80%, with a type I error rate of 5%. Out of the 184 surveyed patients, 161 agreed to participate. These participants filled out the questionnaires online, aided by the medical personnel involved in the study. After receiving 158 completed questionnaires, a total of 149 were selected for the final analysis after excluding any with incomplete medical records.

This research incorporated three different patient groups to assess the influence of age and the severity of craniofacial asymmetry on quality of life and psychosocial impact. Group one included teenagers (14–19 years) with moderate to severe craniofacial asymmetry, while group two consisted of young adult patients (20–25 years) also presenting moderate to severe craniofacial asymmetry. The third group served as a control, encompassing patients aged between 13 and 26 years who showed relative craniofacial asymmetry. All patients were recruited from the same tertiary care hospital, and their diagnoses were substantiated through radiological imaging. The categorization of asymmetry was based on the degree of gnathion deviation, according to the American Association of Orthodontists [17]. Those with a deviation exceeding 2 mm, but not more than 4 mm, were classified as having moderate asymmetry. In contrast, severe asymmetry was attributed to those patients with a gnathion deviation surpassing 4 mm. Relative craniofacial asymmetry was assigned to patients with a gnathion deviation less than 2 mm.

### 2.3. Asymmetry Evaluation

The process of evaluating chin symmetry was conducted by a team of experienced maxillo-facial surgeons and orthodontists. The evaluation incorporated both a clinical examination and a 3D photographic analysis of the patients’ faces. The maxillo-facial surgeons performed the clinical examination, which involved a careful palpation and visual inspection of the patients’ faces, with particular attention paid to the alignment of the gnathion. Thus, 3D photographic analysis was performed by the orthodontists using stereophotogrammetry. This non-invasive technique provided highly accurate 3D images of the patients’ faces. It is worth mentioning that all stereophotogrammetric images were taken with the patients in the natural head position to maintain the standardization of the photographs, as indicated in recent research [18]. This combined approach provided a thorough and accurate evaluation of chin symmetry and overall facial asymmetry.

The displacement of the gnathion away from the midsagittal plane in either the right or left direction was used to establish the direction of mandibular displacement. The angle formed by the intersection of the most anterior and median point of the frontonasal suture with the point located at the largest concavity of the anterior portion of the maxilla (NA) and the most anterior and median point of the frontonasal suture with the point located at the largest concavity of the anterior portion of the mental symphysis (NB) line was used to determine the sagittal jaw relationship of the patient. There were three different categories for the sagittal jaw relationship: Class I (from 0° to 4.5°), Class II (>4.5°), and Class III (0°) [19]. The angle formed by the intersection of the most anterior and median point of the frontonasal suture, the middle point on the anterior rim of the occipital foramen, the most superior and posterior point in the upper contour of the pterygomaxillary fissure, and the most anterior inferior point on the bony chin, were used to determine the vertical skeletal relationship of the patient. The vertical skeletal relationship might be horizontal (more than 93 degrees), normal (between 87 and 93 degrees), or vertical (less than 87 degrees) [20]. The anterior and median points of the frontonasal suture, the point in the center of the sella turcica, and the middle point on the anterior rim of the occipital foramen were used to calculate the cranial base angle. It was determined if the cranial base angle was acute (less than 127 degrees), normal (between 127 and 136 degrees), or obtuse (more than 136 degrees) [21]. Patients were classified as having maxillary asymmetry if the gap between the anterior nasal spine and the midsagittal plane was more than 2 mm. Maxillary asymmetry may either be absent or present [22].

### 2.4. Questionnaires and Variables

The research involved assessing patients’ QoL both before and six months after their initial procedure for craniofacial asymmetry correction, using the questionnaires SF-36, BIQLI, HADS, and PSS-10. The surveys were administered online, and instructions were provided on how to correctly use the scales.

The Short Form-36 (SF-36) is a globally accepted tool for evaluating health-related quality of life (HRQOL) and functional status, widely used in both clinical and research environments. It comprises 36 items, which explore eight domains of HRQOL, including physical functioning, role limitations due to physical health problems, bodily pain, perceptions of general health, vitality, social functioning, role limitations resulting from emotional problems, and mental health. As a self-administered questionnaire, the SF-36 asks respondents to reflect on their health status over the past four weeks. Each domain has a score range from 0 to 100, with higher scores signifying superior health status and quality of life. These domain scores can be combined to generate two summary scores: the Physical Component Summary (PCS) and the Mental Component Summary (MCS). The SF-36 enjoys extensive validation and is accessible in several languages [23].

The Body Image Quality of Life Inventory (BIQLI) is a self-report questionnaire that assesses the impact of self-perceived body image on an individual’s quality of life [24]. The questionnaire consists of 19 items that are divided into three subscales: (1) Satisfaction/Dissatisfaction with Appearance (7 items): this subscale measures the extent to which individuals are satisfied or dissatisfied with various aspects of their appearance, such as their weight, shape, and facial features; (2) Salience of Appearance in Self-Concept (6 items): this subscale measures the extent to which individuals’ self-concept is based on their appearance, such as how much they think about their appearance and how much importance they place on it; and (3) Appearance Investment (6 items): this subscale measures the extent to which individuals invest time, energy, and resources into their appearance, such as through grooming, exercise, and clothing choices. Participants rate each item on a scale from 1 (not at all) to 7 (very much), with higher scores indicating a greater impact of body image on quality of life.

The Hospital Anxiety and Depression Scale (HADS) is a well-accepted self-report scale crafted to gauge anxiety and depression levels in patients undergoing hospital or outpatient treatment [25]. The HADS is composed of 14 items divided into two sections assessing anxiety (HADS-A) and depression (HADS-D), respectively. Each item is rated on a 4-point scale, with higher scores reflecting more intense anxiety or depression. Similarly, the Perceived Stress Scale (PSS-10), another widely accepted self-report tool, quantifies how individuals perceive stressful situations in their lives [26]. It includes 10 items, each rated on a 5-point Likert scale from 0 (never) to 4 (very often). This questionnaire probes various dimensions of perceived stress, including feelings of helplessness and lack of control. The sum of item scores, ranging from 0 to 40, indicates the level of perceived stress, with higher scores signifying increased stress levels. Both the HADS and PSS-10 are recognized for their reliability and validity across different populations and settings.

### 2.5. Statistical Analysis

The software GraphPad Prism (GraphPad, San Diego, California, USA) version 6.0 for Microsoft Windows, produced by GraphPad Software U.S.A., was employed to perform the statistical analysis. To verify the normality of the dataset, we used the Kolmogorov–Smirnov test. For normally distributed data, we employed the mean value, indicating central tendency and standard deviation, signifying data dispersion. The analysis of variance (ANOVA) test served to scrutinize the differences in mean values between the comparison groups. For non-normally distributed data, median values and the interquartile range (IQR) were used as markers, and such data were visualized via box plots. We used the Kruskal–Wallis test to compare these sets of variables. Because the assumptions for the Chi-square test regarding frequency were not met, we employed Fisher’s exact test to compare proportions. The *p*-value was set at less than 0.05 for statistical significance.

## 3. Results

### 3.1. Background Analysis

Table 1 presents the background analysis of the three study groups: teenagers (*n* = 53), adults (*n* = 46), and the control group of patients with a diagnosis of relative craniofacial asymmetry (*n* = 50). The mean age varied significantly among the groups (*p* < 0.001), with teenagers having a mean age of 16.9 ± 2.0 years, adults at 23.6 ± 1.8 years, and the control group at 21.2 ± 4.5 years. Gender distribution was similar across all groups, with females constituting 58.5% of teenagers, 56.5% of adults, and 56.0% of the control group (*p* = 0.964). The participants’ area of residence (urban) was also fairly comparable between the groups (*p* = 0.684). The prevalence of obesity, smoking, and a history of facial trauma did not differ significantly among the three groups (*p* = 0.325, *p* = 0.104, and *p* = 0.717, respectively). When analyzing the asymmetry type, measured by the degree of mandibular displacement, the control group showed 100% relative asymmetry, while the teenagers and adults presented moderate (69.8% and 76.1%, respectively) and severe (30.2% and 23.9%, respectively) asymmetry types, without a statistically significant difference between the two groups (*p* = 0.484).

### 3.2. Craniofacial Characteristics

Table 2 presents the characteristics of craniofacial asymmetry in the study cohort. No significant difference was observed between teenagers and adults in terms of the side of mandibular displacement (*p* = 0.856), with the left side affected in 54.7% of teenagers and 56.5% of adults and the right side in 45.3% and 43.5%, respectively. The sagittal jaw relationship also showed no significant difference between teenagers and adults (*p* = 0.897), with Class I, II, and III relationships distributed similarly among both groups. The vertical skeletal relationship showed no significant difference between teenagers and adults (*p* = 0.363), with horizontal, vertical, and normal relationships similarly distributed among both groups. The cranial base angle, including acute, obtuse, and normal angles, demonstrated no significant difference between teenagers and adults (*p* = 0.841). Lastly, maxillary asymmetry was observed in 15.1% of teenagers, 21.7% of adults, and 14.0% of the control group, with no significant difference between teenagers and adults (*p* = 0.392). Overall, the characteristics of craniofacial asymmetry showed no significant differences between teenagers and adults across the measured variables.

### 3.3. Analysis of Standardized Questionnaires

**Figure 1:**
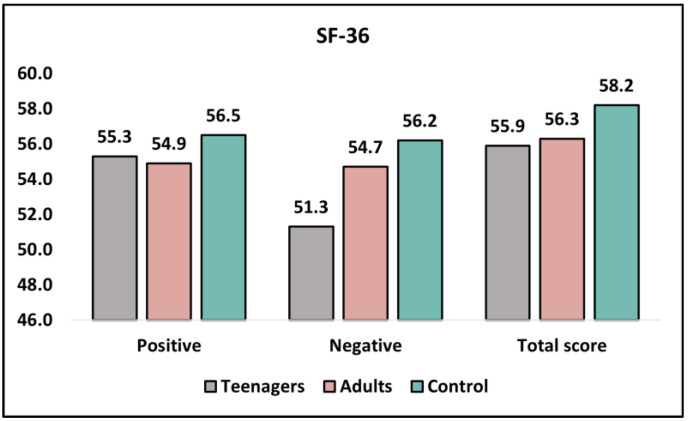
Analysis of SF-36 questionnaire before intervention.

Table 3 and Figure 1 describe the results of the SF-36 questionnaire, comparing teenagers, adult patients, and the control group before intervention and at six months. The SF-36 measures the quality of life across physical and mental health domains, with higher scores representing better outcomes. Before the intervention, the mean physical health scores were similar between teenagers and adults (55.3 ± 7.8 and 54.9 ± 8.0, respectively; *p* = 0.802), with the control group scoring slightly higher (56.5 ± 7.2). However, there was a significant difference in mental health scores between teenagers and adults (51.3 ± 8.4 and 54.7 ± 7.5, respectively; *p* = 0.037), with the control group scoring the highest (56.2 ± 6.9). The total scores before intervention did not differ significantly between teenagers and adults (55.9 ± 8.8 and 56.3 ± 7.9, respectively; *p* = 0.814).

At the 6-month follow-up, the physical health scores for teenagers and adults remained similar (55.6 ± 7.2 and 53.8 ± 8.0, respectively; *p* = 0.241), while the control group had a slightly higher score (57.2 ± 6.8). The mental health scores between teenagers and adults showed no significant difference at this point (53.2 ± 7.9 and 55.6 ± 7.3, respectively; *p* = 0.121), with the control group continuing to score the highest (58.4 ± 7.6). The total scores at six months did not differ significantly between teenagers and adults (56.7 ± 8.0 and 55.9 ± 7.8, respectively; *p* = 0.616).

**Figure 2:**
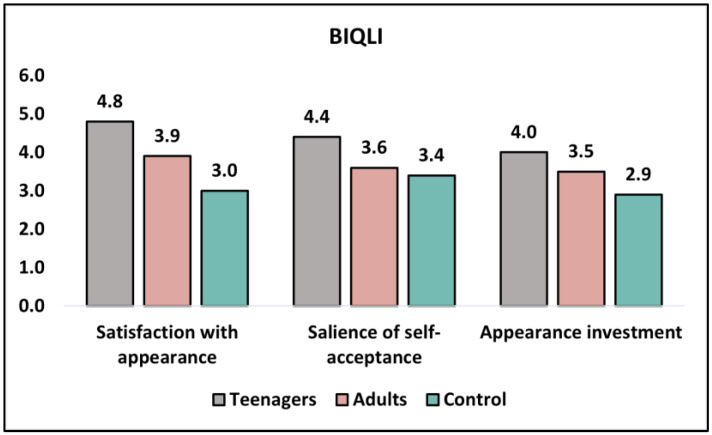
Analysis of the BIQLI questionnaire before intervention.

Table 4 and Figure 2 present the analysis of the BIQLI (Body Image Quality of Life Index) questionnaire results before intervention and at six months. The BIQLI measures satisfaction with appearance, the salience of self-acceptance, and appearance investment, with higher scores indicating worse outcomes. Before the intervention, there was a significant difference in satisfaction with appearance between teenagers and adults (4.8 ± 1.9 and 3.9 ± 2.5, respectively; *p* = 0.045), with the control group having the lowest score (3.0 ± 1.8). The salience of self-acceptance also showed a significant difference between teenagers and adults (4.4 ± 1.8 and 3.6 ± 2.2, respectively; *p* = 0.051), while the control group had the lowest score (3.4 ± 1.5). However, no significant difference was observed in appearance investment between teenagers and adults (4.0 ± 2.1 and 3.5 ± 1.9, respectively; *p* = 0.219), with the control group reporting the lowest score (2.9 ± 2.0).

At the 6-month follow-up, satisfaction with appearance remained significantly different between teenagers and adults (4.1 ± 1.7 and 3.4 ± 2.0, respectively; *p* = 0.062), while the control group continued to score the lowest (2.8 ± 1.8). The salience of self-acceptance showed a significant difference between teenagers and adults (3.8 ± 1.5 and 3.1 ± 1.7, respectively; *p* = 0.031), with the control group reporting the lowest score (2.9 ± 1.9). However, appearance investment scores at six months did not differ significantly between teenagers and adults (2.6 ± 1.9 and 2.5 ± 2.1, respectively; *p* = 0.804), and the control group had a similar score (2.3 ± 1.7).

**Figure 3:**
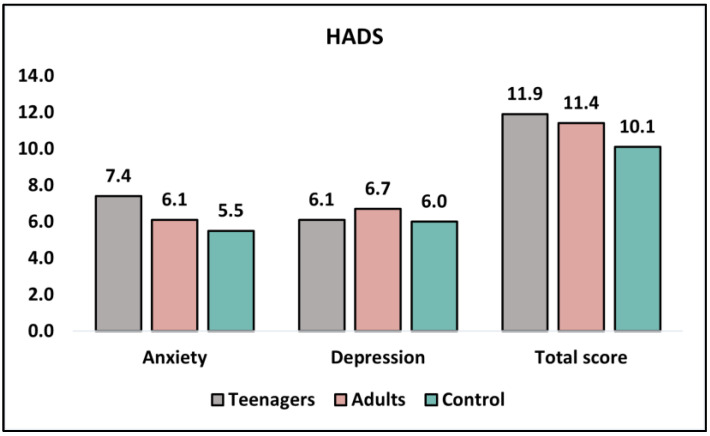
Analysis of the HADS questionnaire before intervention.

Table 5 analyzes the results of the HADS (Hospital Anxiety and Depression Scale) questionnaire before intervention and at six months. The HADS measures anxiety and depression levels, with higher scores indicating greater levels of anxiety or depression. Before the intervention, there was a significant difference in anxiety levels between teenagers and adults (7.4 ± 2.3 and 6.1 ± 3.8, respectively; *p* = 0.039), with the control group reporting the lowest score (5.5 ± 3.2). However, there was no significant difference in depression levels between teenagers and adults (6.1 ± 2.5 and 6.7 ± 2.1, respectively; *p* = 0.203), with the control group scoring similarly (6.0 ± 2.0). The total HADS scores before intervention did not differ significantly between teenagers and adults (11.9 ± 5.0 and 11.4 ± 4.6, respectively; *p* = 0.607), as seen in Figure 3.

At the 6-month follow-up, anxiety levels between teenagers and adults were no longer significantly different (6.9 ± 3.7 and 5.8 ± 3.5, respectively; *p* = 0.133), with the control group reporting a similar score (5.8 ± 3.4). Depression levels remained non-significant between teenagers and adults (5.4 ± 3.1 and 6.1 ± 4.8, respectively; *p* = 0.384), with the control group scoring slightly lower (5.2 ± 3.6). The total HADS scores at six months did not differ significantly between teenagers and adults (10.3 ± 5.5 and 9.9 ± 6.1, respectively; *p* = 0.732).

**Figure 4:**
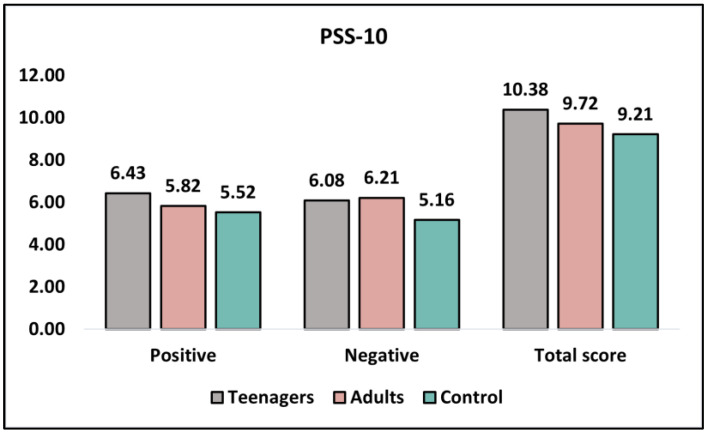
Analysis of the PSS-10 questionnaire before intervention.

Table 6 presents the analysis of the PSS-10 (Perceived Stress Scale) questionnaire before intervention and at six months. The PSS-10 measures positive and negative perceived stress, with higher scores indicating worse outcomes. Before the intervention, there were no significant differences in positive perceived stress between teenagers and adults (6.43 ± 3.12 and 5.82 ± 3.66, respectively; *p* = 0.372), with the control group reporting the lowest score (5.52 ± 2.93). Similarly, negatively perceived stress showed no significant difference between teenagers and adults (6.08 ± 2.89 and 6.21 ± 3.14, respectively; *p* = 0.830), with the control group reporting the lowest score (5.16 ± 2.02). The total PSS-10 scores before intervention did not differ significantly between teenagers and adults (10.38 ± 4.92 and 9.72 ± 4.07, respectively; *p* = 0.473), as presented in Figure 4.

At the 6-month follow-up, positive perceived stress levels remained non-significant between teenagers and adults (6.31 ± 4.13 and 7.06 ± 4.62, respectively; *p* = 0.395), with the control group reporting a similar score (6.01 ± 4.17). Negative perceived stress levels also continued to show no significant difference between teenagers and adults (5.64 ± 3.20 and 5.38 ± 2.91, respectively; *p* = 0.675), with the control group reporting the lowest score (4.83 ± 2.26). The total PSS-10 scores at six months remained non-significant between teenagers and adults (9.59 ± 4.94 and 9.02 ± 5.58, respectively; *p* = 0.591).

## 4. Discussion

### 4.1. Literature Findings

The current study identified significant differences in the quality of life and the psychosocial impact of craniofacial asymmetry in adolescents and young adults undergoing orthodontic and orthognathic correction. The SF-36 questionnaire results revealed no significant differences in physical health scores between teenagers and adults, both before intervention and at six months. However, there was a significant difference in mental health scores before the intervention, which disappeared at the 6-month follow-up. The BIQLI questionnaire results revealed significant differences in satisfaction with appearance and salience of self-acceptance between teenagers and adults before intervention and at six months, while appearance investment did not show significant differences between the two groups at either time point. Also, the HADS questionnaire results revealed a significant difference in anxiety levels between teenagers and adults before the intervention, but this difference disappeared at the 6-month follow-up. Depression levels and total HADS scores showed no significant differences between teenagers and adults at either time point. Lastly, the PSS-10 questionnaire results revealed no significant differences in positive or negative perceived stress levels between teenagers and adults, both before intervention and at the 6-month follow-up. Total PSS-10 scores also showed no significant differences between teenagers and adults at either time point.

The existing literature on craniofacial anomalies highlights social issues, like negative reactions to appearance, coping strategies, struggles with sameness and difference, and the desire for surgery to reduce stigmatization [27]. The need to belong is a fundamental human motivation deeply rooted in our past and crucial for interaction with peers. Threats to connections with others and lack of belongingness are perceived as severe dangers to an individual’s safety and sanity. Our sense of self emerges and is formed through countless interactions with others in various social contexts, starting with attachment relationships with significant others [28]. Belongingness contributes significantly to self-esteem, and our experiences of ourselves are shaped by how we perceive others to feel and think about us.

For people with facial differences, the scrutiny of others’ gazes can be hurtful and damaging to their self-image and self-esteem. They may experience staring, questions, and comments, leading to a feeling of being different and a desire for recognition and acceptance from others [29]. The term “social pain” describes the emotional reaction to feeling excluded and devalued by others, which is painful because social inclusion and belongingness are essential for human survival. Some individuals with facial differences have reported that a lack of belongingness and feeling different from others have significantly impacted their lives, potentially as a result of experiencing social pain [30].

Developed countries value appearance greatly, with body dissatisfaction and concerns about looks being common. Facial differences, whether born with or acquired, can have a significant psychological effect on those who have to deal with an appearance that is noticeably different and stigmatized [31]. The terms “disfigurement” and “deformity” have been replaced with “facial difference” and “visible difference” to reduce stigma. The face is often the first thing we notice about someone and plays a crucial role in forming first impressions, self-expression, communication, social interaction, and understanding others’ feelings. A facial difference can affect one’s self-worth, confidence, and self-esteem, especially if it is around the eyes, mouth, and nose, known as the communication triangle [32]. Those with facial differences may be perceived as less skilled or intelligent and may experience stigmatization. People with facial differences often lack anonymity due to unwanted attention, such as staring, comments, surprise, aversion, or social discomfort, in addition to avoidance and other negative behaviors. Exposure to negative reactions may lead to heightened sensitivity and anticipation of further negative reactions, potentially resulting in low social expectations and social avoidance [33].

The BIQLI scores in our study revealed that teenagers had significantly lower satisfaction with appearance and self-acceptance compared to adults before the intervention. This finding supports the notion that younger patients might be more affected by the aesthetic concerns associated with craniofacial asymmetry, which can negatively influence their body image and self-esteem, as described by other studies that evaluated teenagers’ perception of self when physical factors were involved [34]. Body distortion is a term that is often associated with adolescents, and the BIQLI questionnaire was mostly evaluated in the context of eating disorders (EDs). For example, one study employed the BIQLI and PSQ surveys used in our study and observed that ED patients exhibit the poorest body image quality of life and that the relationship between body image and ED-related variables would be stronger than with other psychological or psychopathological factors, particularly in ED patients [35]. In every aspect of the BIQLI survey, the influence of an unhealthy body image on a person’s quality of life was more detrimental in the ED group.

The HADS questionnaire results further demonstrated that anxiety levels were significantly higher in teenagers compared to adults before the intervention, suggesting that the psychological distress experienced by younger patients might be more pronounced [36]. Another study aimed to determine the prevalence and severity of anxiety and depression in facial palsy patients. More than 100 participants completed the HADS questionnaire, with 32.7% and 31.3% of participants experiencing significant levels of anxiety and depression, respectively [37]. The severity of facial palsy was measured using the House–Brackmann scale, but no significant associations were found between clinical severity and distress levels. However, significant associations were found between participants’ illness perceptions and the level of distress, and females reported significantly higher levels of anxiety than males. The study concluded that the levels of psychological distress were higher than those found in other outpatient attendees. However, it is essential to note that in our study, after six months of treatment, these differences in psychosocial outcomes between teenagers and adults became non-significant, indicating that treatment might help alleviate the psychological burden of craniofacial asymmetry across age groups.

Participants’ motivation for surgery primarily involved the desire for a more normal appearance, to feel less different and reduce negative reactions, similar to findings in other qualitative studies. Motivations for appearance-altering surgery can also include increased self-esteem, self-confidence, better social relationships, reduced negative impact of others’ judgments, and happiness. Interestingly, people without visible differences seeking cosmetic surgery also try to protect themselves from others’ gaze and feel more accepted. The desire to change appearance, whether with or without a visible difference, may reflect underlying psychological needs, like the need to belong and the fear of being perceived as different.

Changing one’s appearance can foster belongingness, feelings of normality, and recognition and acceptance from others. For some individuals, appearance-altering surgery positively impacts their social lives, self-concept, self-esteem, identity, and personality adjustment [38]. However, surgery is not a quick fix for improving quality of life. While some participants were satisfied with surgery outcomes, others experienced profound disappointment when realizing that appearance would never be completely normalized. Therefore, it is likely that people with visible differences often face challenges in social interactions, making friends, and developing long-term relationships. They may experience social anxiety, fear of negative evaluations, unhelpful self-perceptions, low self-esteem, and negative behavior patterns. However, not everyone with a visible difference is equally affected, as individuals handle reactions differently. Factors, such as family support, humor, social skills, determination, self-awareness, and faith, can positively influence coping and adaptation.

Research suggests that subjective experiences, like perceived visibility and social anxiety, are better predictors of psychological distress than objective measures of appearance. For those with severe craniofacial asymmetry, repeated surgical treatment may be necessary throughout childhood and into adulthood [39]. Reconstructive surgery aims to secure adequate function and improve facial symmetry, jaw function, appearance, and dental malocclusion. Treatment plans should be individually tailored and managed by a multidisciplinary craniofacial team. No single surgical protocol exists, and the number of operations depends on the severity of the condition. Age-dependent treatments, with timely interventions at appropriate stages of craniofacial growth and development, increase the chances of a successful outcome. The impact of treatment-related disappointment can be detrimental to those whose expectations are unfulfilled by appearance-altering surgery. Future research should explore the impact and meaning of such disappointments to better understand how to prepare people for treatment and address their psychological needs post-surgery.

Moreover, even though patients in this study were selected based on the gnathion deviation, it is essential to note that facial asymmetries often extend beyond the chin to other anatomical areas, including the mandibular angles [40], which can significantly contribute to a patient’s dissatisfaction with their appearance. The symmetry of the chin concerning the midline of the face has indeed been evaluated in our study; however, it is pertinent to recognize that both defect and excess asymmetries were considered. These two types of asymmetries usually occur at different ages, with defect asymmetries typically appearing earlier than excess asymmetries. This differential timing can have diverse impacts on the emotional development of patients, thus affecting the psychosocial implications of craniofacial asymmetry. Furthermore, our study did not explicitly investigate the presence of a vertical component in facial asymmetry, such as canting of the lips and the occlusal plane, which could potentially affect the overall perception of asymmetry. These aspects represent limitations of our study and highlight the need for further research to comprehensively assess the multi-dimensional nature of craniofacial asymmetry and its psychosocial impacts.

### 4.2. Study Limitations

The current study comes with several limitations, such as the use of convenience sam-pling that could potentially hinder the broader applicability of the results. A more diverse sampling method might have increased the study’s external validity. Additionally, due to the cross-sectional design, we cannot establish causality between the variables studied. Dependence on self-reported measures for assessing patients’ quality of life and persistent symptoms might introduce recall bias, thereby affecting the data’s accuracy and the study’s validity. The study’s execution in a single tertiary care hospital may limit the applicability of findings to other healthcare contexts or geographical regions. Multi-center research could offer more comprehensive insights into age and facial asymmetry complexity impacts. Notably, some patients were omitted due to incomplete medical records, potentially leading to selection bias and affecting the internal validity of the findings. Finally, the reduction in sample size from 184 to 149 due to incomplete records could intro-duce attrition bias, compromising the internal validity of the results. Potential solutions to mitigate these limitations in future research could include a longitudinal study design to establish causal relationships and a multi-center research that could enhance the generalizability of the findings.

## 5. Conclusions

In conclusion, this cross-sectional study revealed that the age and complexity of craniofacial asymmetry influence the quality of life and psychosocial impact in adolescents and young adults undergoing orthodontic and orthognathic correction. The study found no significant differences in craniofacial characteristics between teenagers and adults. However, differences in mental health, satisfaction with appearance, and anxiety levels were observed before the intervention, with teenagers experiencing more negative outcomes. After six months, improvements were seen in the patients’ mental health, body image, and anxiety levels, but no significant differences were found between teenagers and adults. Further longitudinal studies are needed to better understand the long-term effects of these interventions on the quality of life and psychosocial outcomes in patients with craniofacial asymmetry.

## Figures and Tables

**Table 1:** Background analysis.

Variables	Teenagers (*n* = 53)	Adults (*n* = 46)	Control (*n* = 50)	*p*-Value
Age (mean ± SD)	16.9 ± 2.0	23.6 ± 1.8	21.2 ± 4.5	<0.001 ^t^
Age range	14–19	20–25	14–25	-
Gender (female, %)	31 (58.5%)	26 (56.5%)	28 (56.0%)	0.964
Area of residence (urban, %)	33 (62.3%)	26 (56.5%)	27 (54.0%)	0.684
Obesity (yes, %)	4 (7.5%)	8 (17.4%)	6 (12.0%)	0.325
Smoking (yes, %)	3 (5.7%)	9 (19.6%)	8 (16.0%)	0.104
History of facial trauma (yes, %)	6 (11.3%)	6 (13.0%)	4 (8.0%)	0.717
Asymmetry type *				0.484
Relative	0 (0.0%)	0 (0.0%)	50 (100%)	-
Moderate	37 (69.8%)	35 (76.1%)	0 (0.0%)	-
Severe	16 (30.2%)	11 (23.9%)	0 (0.0%)	-

* Measured by degree of mandibular displacement; Data calculated using the Chi-square test or the Fisher’s exact, unless specified differently; ^t^—calculated using the ANOVA test; *p*-value was considered significant for values below 0.05; SD—Standard Deviation.

**Table 2:** Characteristics of craniofacial asymmetry in the study cohort.

Variables (*n*, %)	Teenagers (*n* = 53)	Adults (*n* = 46)	Control (*n* = 50)	*p*-Value	*p*-Value *
Side of mandibular displacement				0.968	0.856
Left	29 (54.7%)	26 (56.5%)	27 (54.0%)		
Right	24 (45.3%)	20 (43.5%)	23 (46.0%)		
Sagittal jaw relationship				0.905	0.897
Class I	22 (41.5%)	17 (37.0%)	22 (44.0%)		
Class II	18 (34.0%)	17 (37.0%)	18 (36.0%)		
Class III	13 (24.5%)	13 (28.3%)	10 (20.0%)		
Vertical skeletal relationship				0.398	0.363
Horizontal	16 (30.2%)	15 (32.6%)	19 (38.0%)		
Vertical	14 (26.4%)	17 (37.0%)	18 (36.0%)		
Normal	23 (43.4%)	14 (30.4%)	13 (26.0%)		
Cranial base angle				0.827	0.841
Acute	17 (32.1%)	14 (30.4%)	16 (32.0%)		
Obtuse	8 (15.1%)	9 (19.6%)	12 (24.0%)		
Normal	28 (52.8%)	23 (50.0%)	22 (44.0%)		
Maxillary asymmetry	8 (15.1%)	10 (21.7%)	7 (14.0%)	0.550	0.392

* Calculated between teenagers and adults; Data calculated using the Chi-square test or the Fisher’s exact, unless specified differently; *p*-value was considered significant for values below 0.05.

**Table 3:** Analysis of SF-36 questionnaire results.

SF-36	Teenagers (*n* = 53)	Adults (*n* = 46)	Control (*n* = 50)	*p*-Value	*p*-Value *
Before intervention					
Physical	55.3 ± 7.8	54.9 ± 8.0	56.5 ± 7.2	0.563	0.802
Mental	51.3 ± 8.4	54.7 ± 7.5	56.2 ± 6.9	0.005	0.037
Total score	55.9 ± 8.8	56.3 ± 7.9	58.2 ± 7.6	0.319	0.814
At 6 months					
Physical	55.6 ± 7.2	53.8 ± 8.0	57.2 ± 6.8	0.079	0.241
Mental	53.2 ± 7.9	55.6 ± 7.3	58.4 ± 7.6	0.003	0.121
Total score	56.7 ± 8.0	55.9 ± 7.8	57.7 ± 7.5	0.524	0.616

Data calculated using the ANOVA test; *p*-value was considered significant for values below 0.05; * Calculated between teenagers and adults; SF-36—36-Item Short Form Survey; Higher scores represent better outcomes.

**Table 4:** Analysis of BIQLI questionnaire results.

BIQLI	Teenagers (*n* = 53)	Adults (*n* = 46)	Control (*n* = 50)	*p*-Value	*p*-Value *
Before intervention					
Satisfaction with appearance	4.8 ± 1.9	3.9 ± 2.5	3.0 ± 1.8	<0.001	0.045
Salience of self-acceptance	4.4 ± 1.8	3.6 ± 2.2	3.4 ± 1.5	0.018	0.051
Appearance investment	4.0 ± 2.1	3.5 ± 1.9	2.9 ± 2.0	0.023	0.219
At 6 months					
Satisfaction with appearance	4.1 ± 1.7	3.4 ± 2.0	2.8 ± 1.8	0.002	0.062
Salience of self-acceptance	3.8 ± 1.5	3.1 ± 1.7	2.9 ± 1.9	0.021	0.031
Appearance investment	2.6 ± 1.9	2.5 ± 2.1	2.3 ± 1.7	0.719	0.804

Data calculated using the ANOVA test; *p*-value was considered significant for values below 0.05; * Calculated between teenagers and adults; BQLI—The Body Image Quality of Life Index; SD—Standard Deviation; IQR—Interquartile Range; Higher scores represent worse outcomes.

**Table 5:** Analysis of HADS questionnaire results.

HADS	Teenagers (*n* = 53)	Adults (*n* = 46)	Control (*n* = 50)	*p*-Value	*p*-Value *
Before intervention					
Anxiety	7.4 ± 2.3	6.1 ± 3.8	5.5 ± 3.2	0.008	0.039
Depression	6.1 ± 2.5	6.7 ± 2.1	6.0 ± 2.0	0.254	0.203
Total score	11.9 ± 5.0	11.4 ± 4.6	10.1 ± 4.3	0.135	0.607
At 6 months					
Anxiety	6.9 ± 3.7	5.8 ± 3.5	5.8 ± 3.4	0.195	0.133
Depression	5.4 ± 3.1	6.1 ± 4.8	5.2 ± 3.6	0.491	0.384
Total score	10.3 ± 5.5	9.9 ± 6.1	9.1 ± 5.2	0.546	0.732

Data calculated using the ANOVA test; *p*-value was considered significant for values below 0.05; * Calculated between teenagers and adults; HADS—Hospital Anxiety and Depression Scale; Higher scores indicate greater levels of anxiety or depression.

**Table 6:** Analysis of the PSS-10 questionnaire results.

PSS-10	Teenagers (*n* = 53)	Adults (*n* = 46)	Control (*n* = 50)	*p*-Value	*p*-Value *
Before intervention					
Positive	6.43 ± 3.12	5.82 ± 3.66	5.52 ± 2.93	0.349	0.372
Negative	6.08 ± 2.89	6.21 ± 3.14	5.16 ± 2.02	0.115	0.830
Total score	10.38 ± 4.92	9.72 ± 4.07	9.21 ± 5.33	0.468	0.473
At 6 months					
Positive	6.31 ± 4.13	7.06 ± 4.62	6.01 ± 4.17	0.473	0.395
Negative	5.64 ± 3.20	5.38 ± 2.91	4.83 ± 2.26	0.337	0.675
Total score	9.59 ± 4.94	9.02 ± 5.58	8.27 ± 5.12	0.437	0.591

Data calculated using the ANOVA test; *p*-value was considered significant for values below 0.05; * Calculated between teenagers and adults; PSS-10—The Perceived Stress Scale; Higher scores represent worse outcomes.

## Data Availability

Data available on request.
